# Characterizing injury at a tertiary referral hospital in Kenya

**DOI:** 10.1371/journal.pone.0220179

**Published:** 2019-07-24

**Authors:** Hannah Janeway, Gerard O’Reilly, Florian Schmachtenberg, Nimai Kharva, Benjamin Wachira

**Affiliations:** 1 Harbor-UCLA Medical Center, Torrance, California, United States of America; 2 Emergency and Trauma Centre, The Alfred, Melbourne, Australia; 3 School of Public Health and Preventive Medicine, Monash University, Melbourne, Australia; 4 Accident & Emergency Department, The Aga Khan University Hospital, Nairobi, Kenya; Tsinghua University, CHINA

## Abstract

**Introduction:**

Injury accounts for more than 5.8 million deaths globally with an increasing burden in the developing world. In Kenya, trauma is one of the top 10 leading causes of death. However, no formal continuous injury surveillance systems are in place to inform injury prevention, pre-hospital care or emergency department management. The aim of this study was to implement a hospital-based trauma registry to characterize high acuity injuries presenting to a private tertiary, teaching and referral hospital in Kenya.

**Methods:**

From January to December 2015, data was prospectively collected at a private tertiary, teaching and referral hospital in Nairobi, Kenya. Patients presenting with a traumatic injury for the first time who were admitted to the hospital for at least 48 hours were included in the study. Basic information pertaining to demographics, details of the injury, pre-hospital care and transport, hospital-based management, length of stay and disposition were collected. An injury severity score (ISS) was calculated on each patient and stratified by the mechanism of injury. Descriptive statistics and multivariate logistic regression were used to analyze data and assess risk factors associated with injury severity.

**Results:**

There were 101 patients included in the study, the majority of whom were 30 to 39 years of age and male (63%). Seventy-one per cent of patients had a preexisting medical condition with hypertension (26%) and diabetes (13%) being the most common. The most common mechanism of injury was fall (46%) followed by road traffic incidents (RTI) (32%). Most injuries took place at home (43%). Most RTI were caused by cars (63%), with the driver being the most frequently injured (38%). The most common mode of arrival to the emergency department was by private car (72%). The median time between the accident and arrival at the emergency department was 1hr 10 minutes. The majority of the patients had injuries to one area (83%) with the extremities/bony pelvis (72%) being the most common. The median Injury Severity Score was 5 (range 1–34) with the majority (90%) classified as minor injuries (ISS<12). The highest severity of injury as determined by ISS was seen in gunshot wounds.

**Conclusions:**

Injured patients in Kenya showed concordance with prior studies looking at injury prevalence in the developing world when looking at demographics and place of injury. However, differences were found when looking at the mechanism of injury, with falls surpassing road traffic incidents. A delayed presentation to the hospital was also noted in this patient population. Given the rate of traumatic injuries in Kenya and their contribution to morbidity, mortality and overall healthcare costs, there is a need to implement formal trauma registries in all major hospitals in Kenya to generate more data that can be used to improve injury prevention, the overall trauma system and enhance training and preparedness.

## Introduction

Injury accounts for more than 5.8 million deaths globally and is increasingly becoming one of the highest contributors to morbidity and mortality in the developing world. [1] This is approximately 10% of mortality globally and 32% more deaths than malaria, tuberculosis and HIV/AIDs combined. [2] In 2010, 90% of injured patients were found in low and middle-income countries (LMICs). [3] Individuals in LMICs are six times more likely to die from trauma than those in high-income countries. [4] According to the World Health Organization (WHO), the burden of disease related to injuries, particularly road traffic injuries, interpersonal violence, war and self-inflicted injuries is expected to rise dramatically by the year 2020. [5]

In Kenya, trauma is one of the top 10 leading causes of death. [6] Despite the increasing burden of injury, there are no continuous trauma registries in Kenya for collection and analysis of injury data, which limits the understanding and improvement of trauma care. Trauma-related data is scarce and trauma documentation is not standardized. Pre-hospital data is rudimentary and comes mostly from the police, Emergency Medical Services (EMS) and the National Transport and Safety Authority (NTSA). Some information can be gleaned by reviewing the medical chart, but it is often incomplete and does not provide key details that are necessary for formulating a comprehensive approach to improving overall care for the acutely injured patient.

In many countries, formal trauma registries have been implemented to address these issues. A trauma registry is a hospital-based collection of uniform data describing individuals who are injured in which clinical, demographic and outcome data are documented in an ongoing and systematic manner. [7] This data can then be used both by public health officials seeking to improve injury prevention, pre-hospital care and overall trauma systems and by local hospital officials to enhance training and preparedness.

The primary aim of this study was to implement a hospital-based trauma registry for high acuity injuries and to gather data pertaining to the characteristics of injury and the care provided to injured patients, including at the pre-hospital level, at a private tertiary, teaching and referral hospital in Kenya. Secondary aims included determining the feasibility of introducing data collection into a large metropolitan hospital in sub-Saharan Africa and an analysis of risk factors for severe injury.

## Methods

The study was conducted at the Aga Khan University Hospital, Nairobi (AKUH,N), which is a private tertiary, teaching and referral hospital that serves as a post-graduate teaching hospital. The Emergency Department (ED) sees approximately 70,000 patients annually, with a 10–15% admission rate. [8]

A trauma registry data collection sheet was developed prior to study implementation by surveying physicians working in the AKUH,N ED, reviewing publicly available injury surveillance collection techniques employed worldwide, and through consideration of details important to pre-hospital, emergency department and inpatient care. The registry was designed to collect data related to demographics, injury history, pre-hospital care, diagnoses, injury severity, management, disposition and outcomes.

The study was conducted prospectively during a period of 12 months from January 2015 to December 2015. This period of time (one calendar year) was chosen for pragmatic reasons, to ensure the organizational commitment to the feasibility of data collection for this new registry. Patients presenting to the ED immediately following an injury and admitted to the hospital for at least 48 hours were included in the study. Patients who were dead on arrival or patients who died during evaluation or treatment in the ED were also included. Exclusion criteria included pediatric patients (<15 years) as they presented to the pediatric emergency department, patients who transferred in from another facility or transferred out of the hospital, patients seen and discharged from the ED, isolated hip fractures, and patients presenting with delayed injury over 24hrs.

The trauma registry sheet was completed by the ED doctor after stabilization of the patient. Trauma admissions were reviewed daily, and missing data in the trauma registry sheets were completed by study coordinators via chart review and patient interview prior to discharge.

Descriptive statistics were generated to analyze the data from the trauma registry and provide a profile of the injured patients. The Injury Severity Score (ISS) was calculated for all the patients. Minor injury was classified as ISS ≤ 12 and major injury as >12 per AIS 2005.

To determine the crude risk factors for major injury (ISS>12), univariable logistic regression was performed. Then, to determine the independent risk factors for major injury, multivariable logistic regression was conducted, including those variables for with the p-value was <0.05 on univariable logistic regression.

To determine the crude risk factors for a greater hospital length of stay (more than 7 days), univariable logistic regression was performed. Then, to determine the independent risk factors for a greater hospital length of stay, multivariable logistic regression was conducted, including those variables for with the p-value was <0.05 on univariable logistic regression.

Data was analyzed using Microsoft Excel 2011, R version 3.3.1 [9] and Stata version 15.1.

Numerical data was described using mean (standard deviation) if symmetrical and median (range) if non-normal; categorical data was described using raw numbers (%). For comparing means, medians and proportions, the t-test, rank-sum test and chi-square (or Fisher’s Exact test) were used, respectively to test for statistical significance (p<0.05).

The study was submitted and approved by the Research Ethics Committee at the Aga Khan University, Nairobi. The Research Ethics Committee waived the need for informed consent as there was no patient contact and no patient identification data collected.

## Results

### Demographics

During the study period, a total of 1,106 adult patients presented with injuries and were admitted to the hospital. Of those, 101 were included in the study per inclusion and exclusion criteria and characteristics of injury recorded (see Table 1).

**Table 1:** Patient characteristics.

Characteristic		
**Numerical variables** **(symmetrical data)**	**Mean**	**SD**
First ED breathing rate (/min)	18	3
First ED SpO2	96	10
First ED pulse rate (/min)	88	15
First ED SBP (mmHg)	134	25
**Skewed numerical or ordinal data**	**Median**	**Range**
Age (years)	42	18–91
Prehospital time—injury to ED (min)	70	5–2860
First ED GCS total	15	9–15
Injury Severity Score	5	1–34
Hospital length of stay (days)	5	1–36
**Categorical variables**	**Number (n = 101)**	**Percent (%)**
**Age groups**		
10–19	5	5.0%
20–29	11	10.9%
30–39	28	27.7%
40–49	20	19.8%
50–59	12	11.9%
60–69	11	10.9%
70–79	8	7.9%
80–89	5	5.0%
90–99	1	1.0%
**Gender**		
Male	64	63.4%
Female	37	36.6%
**Mode of Arrival**		
Private Car	73	72.3%
Ground Ambulance	23	22.8%
Fixed-wing Ambulance	1	1.0%
Taxi	1	1.0%
Foot	2	2.0%
Helicopter Ambulance	2	2.0%
**Pre-arrival Interventions**		
None	81	80.2%
C-Collar	1	1.0%
Splinting	11	10.9%
Analgesia	10	9.9%
IV Access	3	3.0%
Reduction of deformity	1	1.0%
Wound Dressing	4	4.0%
Oxygen	1	
**Places of Injury**		
Athletics Area	3	3.0%
Open land/Beach/Forest/Desert	4	4.0%
Other	1	1.0%
Private Home	43	42.6%
Public Administrative Area	7	6.9%
Street/Highway/Road	34	33.7%
Industrial Area	7	6.9%
Unknown	2	2.0%
**Activity during Trauma**		
Leisure	41	40.6%
Other Work (non-income)	5	5.0%
Sports	4	4.0%
Travelling	26	25.7%
Unknown	1	1.0%
Activities of Daily Life	14	13.9%
Income Generating Work	10	9.9%
**Intent of Injury**		
Accidental	84	83.2%
Intentional (Assault)	16	15.8%
Intentional (Self Harm)	1	1.0%
**Mechanism of Injury**		
Assault with a blunt force object	8	7.9%
Burns	1	1.0%
Crush Injury	1	1.0%
Electrocution	1	1.0%
Fall	46	45.5%
Gun shot wound	8	7.9%
Road Traffic Incident	32	31.7%
Stab wound	4	4.0%
**Injury Severity Score**		
Minor (< 12)	91	90.0%
Major (>12)	10	10.0%
**Disposition**		
HDU	9	8.9%
Ward	78	77.2%
Operating Theatre	12	11.9%
ICU	3	3.0%
**Hospital Discharge Disposition**		
Died in Hospital	3	3.0%
Home	97	96.0%
Left Against Medical Advice	1	1.0%
**Number of anatomic systems injured**		
1 System	83	82.2%
2 Systems	16	15.8%
3 Systems	2	2.0%
**Distribution by anatomic region**		
Extremities including bony pelvis	78	77.2%
Abdominal and pelvic contents	1	1.0%
Face	9	8.9%
Chest	4	4.0%
Head or Neck	25	24.8%
Back	2	2.0%

**Fig 1:**
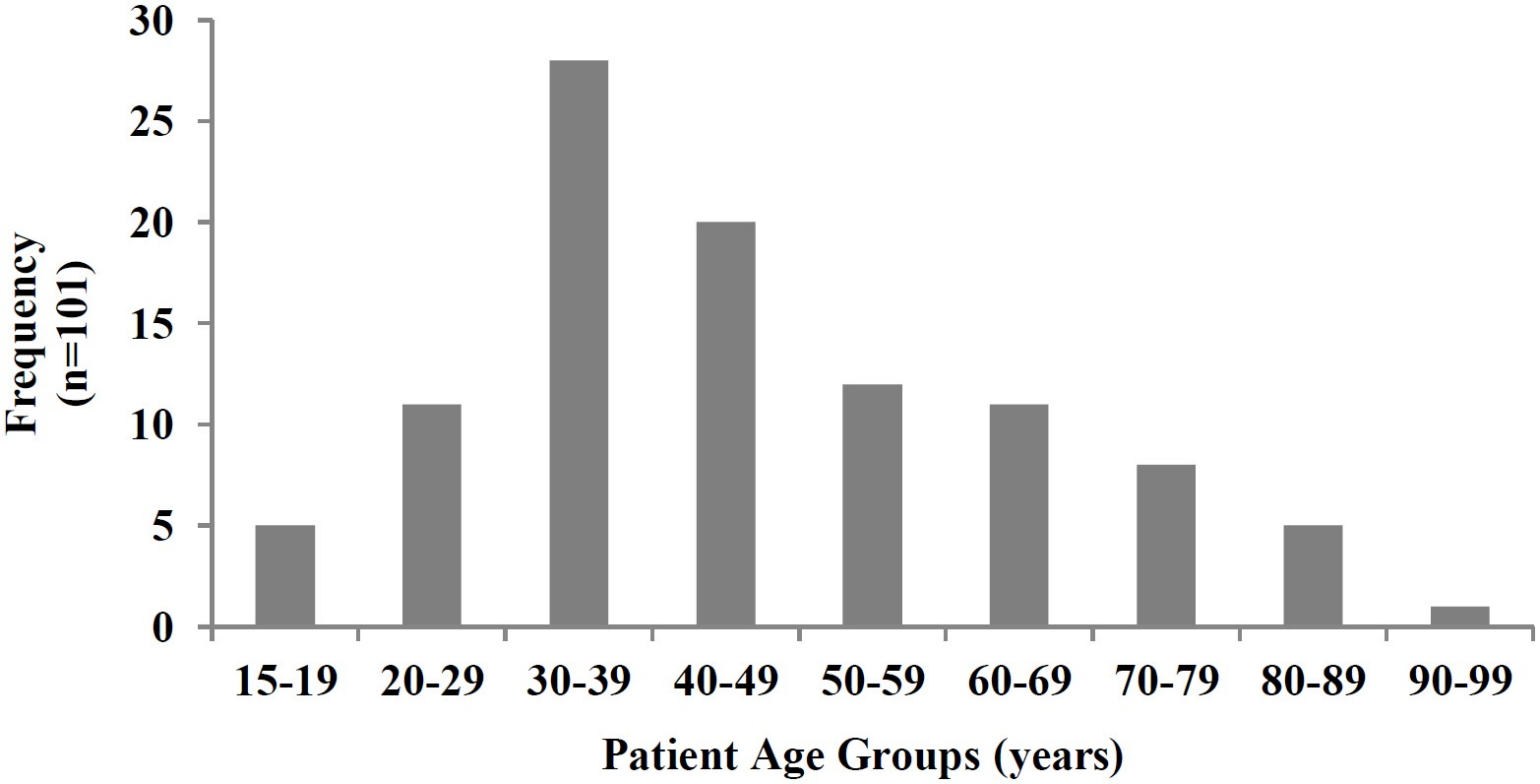
Age distribution of patients.

Fig 1 presents the age distribution of the patients included in the study. The median age of patients was 42 (range 18–91), with the majority falling between 30–39. The majority (63%, n = 64) were male.

Seventy-one percent of patients had a preexisting medical condition. The most common comorbid conditions were hypertension (26%), Diabetes (13%) and Asthma (5%). Four percent of patients had HIV or dyslipidemia.

### Events surrounding injury

**Fig 2:**
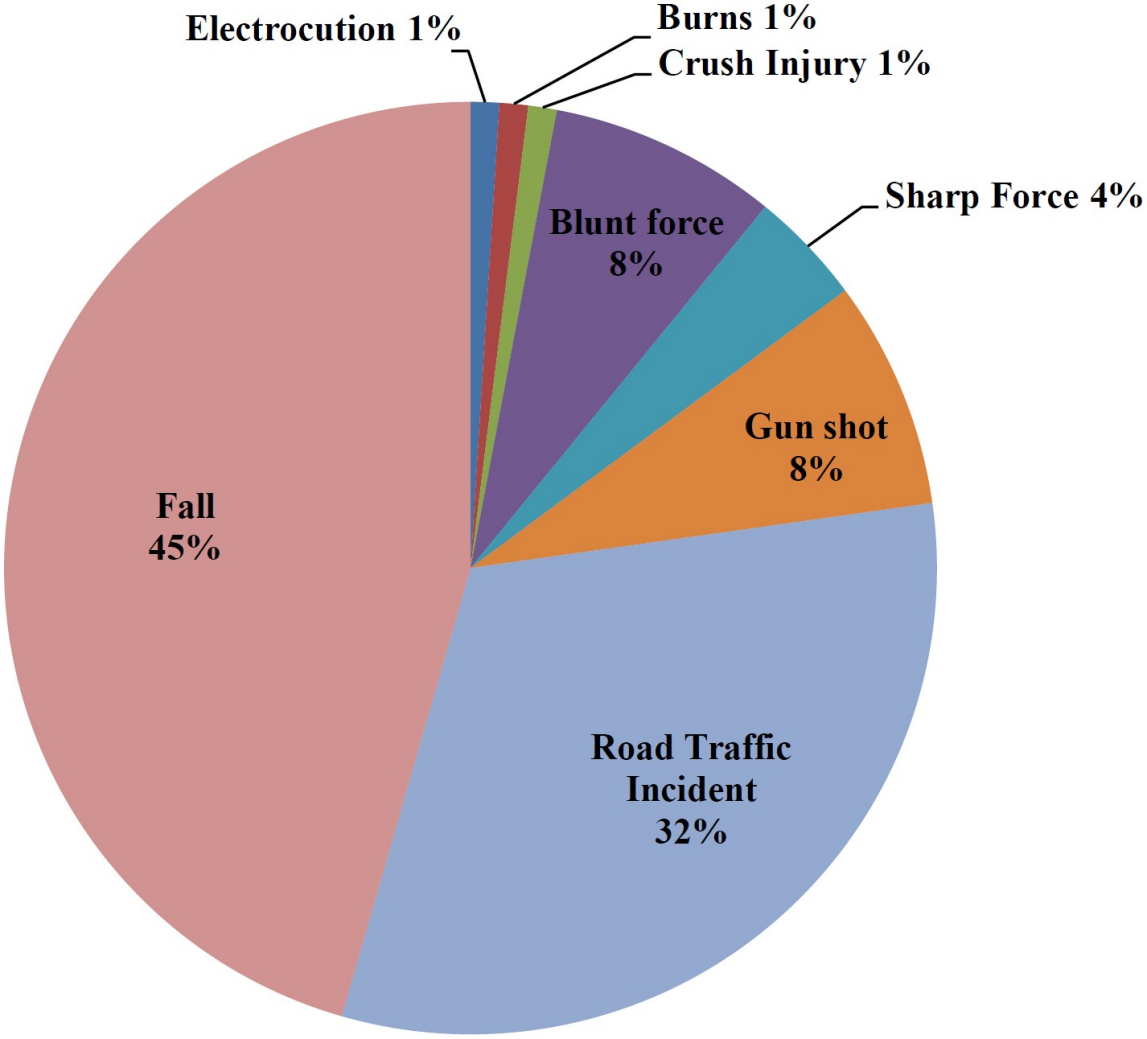
Mechanism of injury of patients.

Fig 2 presents the percentage of patients in the study by the mechanism of injury. The most common mechanism of injury was fall (46%) followed by road traffic incidents (32%). Assault with a blunt force object and gunshot wounds comprised 8% each, with stab wounds only 4%

Eighty-three percent were accidental, 16% intentional-assaults and 1% intentional-self harm. Most injuries took place in private homes (43%), whereas 34% occurred on street/highway/roads, 7% in public administrative area and industrial areas each, 4% in open land/beach/forest/desert, and 3% in areas specified for sport. The most common activities being practised at the time of trauma were leisure (41%) and travelling (26%). Work-related events only occurred around 15% of the time.

Of the 101 patients included in the study, 32% were victims of road traffic incidents (RTI). The majority of the incidents were secondary to car accidents (63%) followed by motorcycles (25%). Matatus (minibuses) comprised only 9% and buses 3% of RTIs.

The most commonly injured person was the driver/rider (37%), followed by pedestrians (28%) and the back-seat passenger (37%). A little over a third (36%) of people involved in a RTI (auto vs pedestrian excluded) had impact protection (e.g. airbags, seatbelts, helmet). Only 46% of people used seatbelts. Half of the motorcyclists injured were un-helmeted.

### Pre-hospital care

Table 2 presents the percentage of included patients by mode of arrival to the ED. The most common mode of arrival was a private vehicle (73%). One-quarter of people (26%) arrived via ground or air ambulance, and 2% arrived on foot.

**Table 2:** Time to arrival by mode of arrival.

Mode of Arrival	Pre-Hospital (%)	Time to Arrival Median (Range)
Private Car/Taxi	3%	1:00 (0:10–23:15)
Ground Ambulance	23%	1:30 (0:05–17:45)
Air Ambulance	3%	4:00 (0:20–23:40)
Foot	71%	3:25 (1:20–5:30)

The median time between the accident and arrival at the emergency department was 1hr 10 minutes (range 00:10–47:40). Ground ambulances took on average 2 hours 49 minutes, with a median of 01:30 (00:35–17:45). The median time for private cars/taxis took 1hr (range 00:10–23:15). The median for helicopter/fixed wing air transport was 4hrs (00:20–23:40).

Of the 101 patients included, only 21 (20%) had pre-hospital interventions. Common pre-hospital interventions done by ambulance crew included analgesia (30% of ambulance runs), splinting (25%) and intravenous access (8%). Pre-hospital interventions were also performed by bystanders and included splinting (5% of non-ambulance transport) and analgesia (3%).

### Injury characteristics and management

Patient injuries were classified into different major anatomical regions via the trauma registry sheet: extremities including the bony pelvis, abdomen/pelvic contents, face, chest, head and neck, and back. Most patients were injured in only one (83%) or two (16%) anatomic regions. The remaining 3% had injuries to three or more locations. The most common location of injury overall were the extremities or bony pelvis (78%), followed by head and neck (25%) and face (9%).

**Fig 3:**
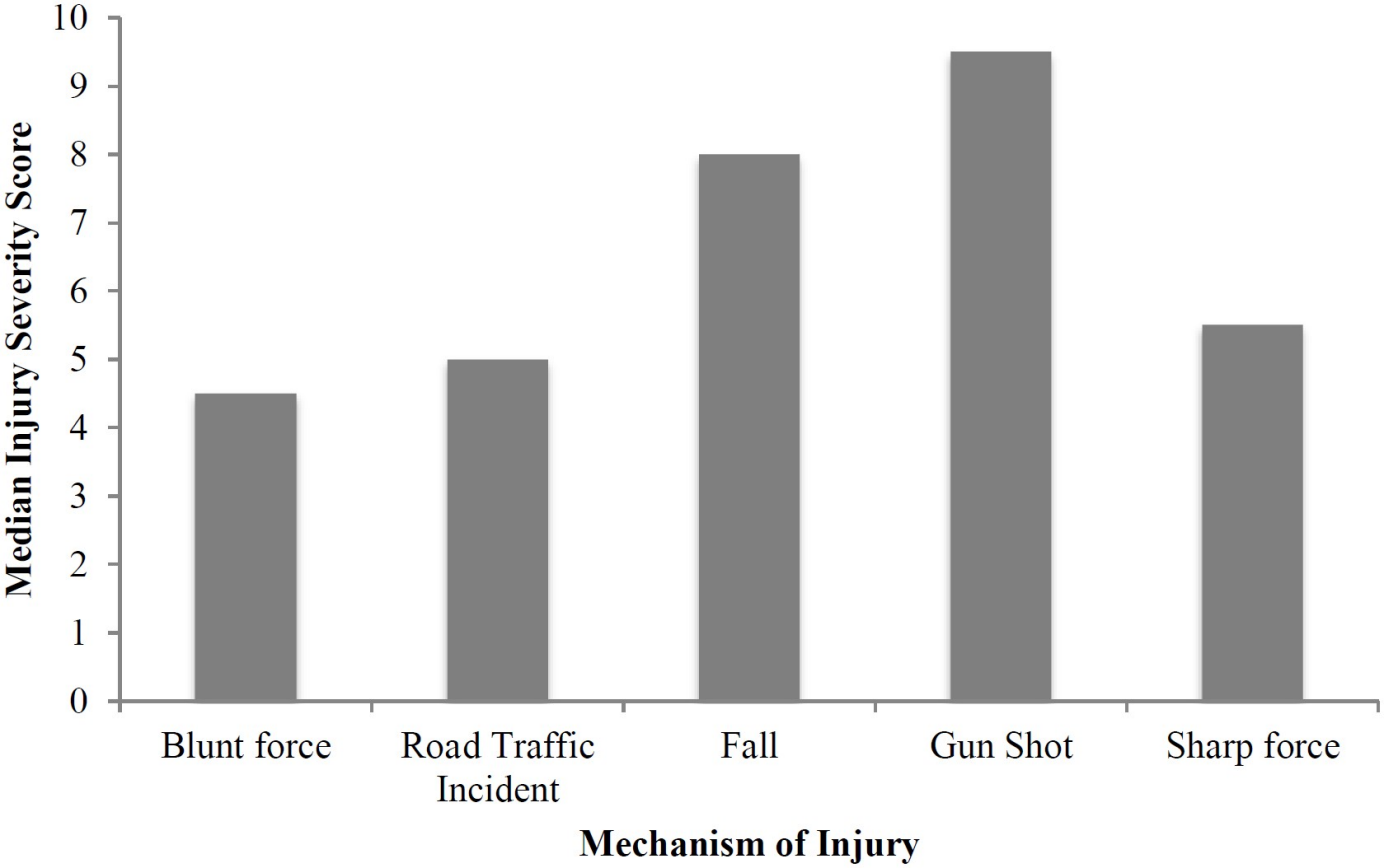
Median ISS by the mechanism of injury.

Fig 3 presents the association between Injury Severity Score and the mechanism of injury. The median Injury Severity Score was 5 and the proportion with major trauma was 10% using an ISS of >12. Looking at the different mechanisms of injury, gunshot wounds had the highest median ISS score followed by falls (See Fig 3). Gunshot wounds had the highest median ISS at 9.5.

Most patients (77%) were admitted to the ward. Nine patients were admitted to the High Dependency Unit (HDU) and 3 patients to the Intensive Care Unit (ICU). Twelve (12%) patients went directly to the operating room from the emergency department.

Ninety-six percent of patients survived to hospital discharge, 3% died and one patient left against medical advice.

The results of the analysis of risk factors for major injury (ISS>12) are provided in Table 3. Following multivariable logistic regression, the independent risk factors for major injury (ISS>12) were determined to be if the injury was intentional and if the first hospital GCS was abnormal.

**Table 3:** Association (risk factors) between injury severity and measured exposure variables.

Exposure variables	ISS>12 (n = 11)	ISS<12 (n = 90)	Univariable		Multivariable	
			OR (95%CI)	p-value	OR (95%CI)	p-value
Age (>65y)	2 (18%)	18 (20%)	0.9 (0.2–4.5)	0.89		
Ambulance to hospital (vs non-ambulance)	5 (45%)	21 (23%)	2.7 (0.8–9.9)	0.12		
Fall (mechanism)	5 (45%)	41 (46%)	1.0 (0.3–3.5)	1.00		
Home (place)	7 (64%)	36 (40%)	2.6 (0.7–9.6)	0.15		
Leisure (activity)	6 (55%)	35 (39%)	1.9 (0.5–6.7)	0.32		
Intent (accidental)	6 (55%)	78 (87%)	0.2 (0.0–0.7)	0.01	0.15 (0.0–0.7)	0.02
Systolic blood pressure <90mmHg	1 (9%)	2 (2%)	4.4 (0.4–53.0)	0.24		
Heart rate >120/min	1 (9%)	1 (1%)	8.9 (0.5–153.5)	0.13		
GCS total <15	5 (45%)	2 (2%)	36.7 (5.8–230.1)	<0.001	45.6 (6.1–340.4)	<0.001

The results of the analysis for an association between a greater hospital length of stay (more than 7 days) and major injury are provided in Table 4. Following multivariable logistic regression, it was determined that a greater hospital length of stay was independently associated with major injury (ISS>12) (p = 0.02).

**Table 4:** Association between hospital length of stay (LOS) and injury severity.

Exposure variables	Hospital LOS>7 days (n = 20)	Hospital LOS< = 7 days (n = 81)	Univariable		Multivariable	
			OR (95%CI)	p-value	OR (95%CI)	p-value
Injury Severity—major (ISS>12)	5 (25%)	6 (7%)	4.2 (1.1–15.4)	0.03	5.4 (1.3–22.4)	0.02
Age (>65y)	8 (40%)	12 (15%))	3.8 (1.3–11.3)	0.02	2.6 (0.7–9.3)	0.16
At least one comorbidity	13 (65%)	29 (36%)	3.3 (1.2–9.3)	0.02	2.6 (0.7–8.8)	0.14
Ambulance to hospital (vs non-ambulance)	7 (35%)	19 (23%)	1.8 (0.6–5.0)	0.29		
Fall (mechanism)	9 (45%)	37 (46%)	1.0 (0.4–2.6)	0.96		
Home (place)	8 (40%)	35 (43%)	0.9 (0.3–2.4)	0.80		
Leisure (activity)	7 (35%)	34 (42%)	0.7 (0.3–2.1)	0.57		
Intent (accidental)	17 (85%)	67 (83%)	1.2 (0.3–4.6)	0.80		
Systolic blood pressure<90mmHg	1 (5%)	2 (2%)	2.1 (0.2–24.1)	0.56		
GCS total<15	2 (10%)	5 (6%)	1.7 (0.3–9.4)	0.56		
Prehospital time>60min	10 (50%)	45 (56%)	0.8 (0.3–2.1)	0.66		
Prehospital interventions performed	7 (35%)	14 (17%)	2.6 (0.9–7.6)	0.09		

## Discussion

Data collected during the study course shows concordance with many of the published studies of injury surveillance both nationally and internationally. Males were predominantly the victims of trauma, likely because of the higher rate of risk-taking activities in daily life. As noted, the majority of our patients were injured during leisure time. In addition, we found the average age of the injured patient to be similar to prior studies, resting in the middle of the economically active population. Our study did not look into the overall morbidity associated with such injuries and more research will be needed to show the likely devastating overall economic impact of such injuries on the Kenyan economy.

Our data also illustrated the limited nature of pre-hospital care in Kenya. The median time to care was 1:10h with ground ambulances taking longer than private cars. The majority had no pre-hospital interventions. It is unclear why private cars arrived more expeditiously to the hospital, however it is likely due to the limited and underdeveloped nature of pre-hospital care in Kenya. Some patients received pre-hospital care from bystanders instead of EMS personnel. The large proportion of individuals who are being transported in private cars or taxis illustrates the potential benefits of public health campaigns focusing on bystander first aid. Programs such as Stop the Bleed that teach civilians to perform potentially life-threatening actions could theoretically greatly decrease in and out of hospital mortality.

It is important to compare our results to a recently completed study at a public referral hospital in Nairobi. Botchey et al illustrated success with a tablet-based method of collection for injuries and reported findings captured in this electronic database. [10] Data coincided in some areas such as gender (male predominance), lack of formal pre-hospital transport (private car/taxi), and age generally. However, there were key differences as well including the mechanism of injury. At the public hospital, RTIs predominated whereas in our population falls were the most frequent. In addition, for those who did present with RTIs, they were most commonly drivers of private vehicles, which differed from the auto vs pedestrian predominance was seen by Botchey et al. Similarly, a study on injury fatalities in Nairobi showed most injury deaths at the largest public mortuary were secondary to RTIs, followed by GSW and assault. Only 1.7% came from falls. [11] It is possible that these differences arise from our inclusion criteria that allowed only individuals who were admitted for >48hrs to be included but could also be secondary to the difference in demographics of those visiting public vs private hospitals. In addition, a much longer time to presentation of care was seen in our patient population than at the public hospital where they observed that over 50% of patients arrived within one hour of injury.

As have been seen in other studies in Kenya, the use of helmets and other protective mechanisms in this study was low. This is consistent with observational studies done in the developing world and in Kenya itself. However, the rate of helmet use in other African countries such as Tanzania (22.7%) [12] and Nigeria (8.1%) [13] was notably less than observed in this study. In Kenya, it is unclear why helmet use continues to be around 50% or less, as noted in our study and others, despite a helmet law that was enacted in 2009, but factors could include the price of helmets, poor understanding of their benefit and use, and the fact that helmets are not yet part of common practice in Kenya. This is a potential area of injury research and increased injury prevention campaigns. Seatbelts were used even less (45%), despite the seatbelt laws enacted in 2013. Despite the fact that less than 50% of patients utilized seat belts, this was considerably higher than prevalence found in South Africa (25.2%), Nigeria (18.7%), or Zimbabwe (46.9). [14],[15],[16] Hospital-based programs aimed at reinforcing these protective measures could be beneficial and are an area of potential future research.

The study has obvious limitations, the most obvious of which is selection bias given that data was collected at a single centre in Nairobi. Aga Khan is a private hospital and while all patients despite their economic means are treated in the emergency department, many patients of lower socioeconomic status will preferentially choose a public hospital for their care as it is more affordable. In addition, Aga Khan is located in an area with a higher socioeconomic status and thus is more likely to be a care centre for patients from this economic group. Second, given the lack of pre-hospital care, it is likely that many of the high acuity traumatic injuries that we were attempting to enrol did not survive to the hospital. Given we did not include any data surrounding pre-hospital deaths, the data is likely biased to those individuals with less life-threatening emergencies. Lastly, our sample size was relatively small, potentially limiting the capacity to perform detail comparative analyses. Notwithstanding this limitation, the study still provides an important insight into the characteristics of injury presenting to a large metropolitan private hospital over the course of one year, consistent with the primary aim of the study.

This study raises many questions about the nature of the injured patient presenting to private hospitals in Kenya. A larger scale trauma registry looking at both public and private settings, rural and urban, would allow comparisons to be made and improve overall hospital preparedness. It will also help direct resources and trauma training efforts to those areas with the high incidence and acuity of injury. Pre-hospital care also remains understudied. The prolonged transportation times seen in this study should be investigated to look for appropriate interventions. Lastly, more in-depth research into the underutilization of preventative measures (e.g. helmets, seatbelts) could help inform large-scale injury prevention efforts.

## Conclusion

Injured patients in Kenya showed concordance with prior studies looking at injury prevalence in the developing world when looking at general demographics and place of injury. However, differences were found especially when looking at the mechanism of injury and transportation time to the hospital. Given the rate of traumatic injuries in Kenya and their contribution to morbidity, mortality and overall healthcare costs, there is a need to implement formal trauma registries in all major hospitals in Kenya to generate more data that can be used to improve injury prevention, the overall trauma system and enhance training and preparedness.

## Supporting information

S1 File Trauma registry data collection sheet. (DOCX)

S2 File Data set. (XLSX)
