# Supplementary material for: Characterizing injury at a tertiary referral hospital in Kenya
Source: PLoS One. 2019 Jul 24;14(7):e0220179. doi: 10.1371/journal.pone.0220179 (PMC6655740; doi:10.1371/journal.pone.0220179)
Supplement: S1 File — (DOCX) [file pone.0220179.s001.docx]

| **Trauma Registry Form** | | | | | | | | | | | | |
| --- | --- | --- | --- | --- | --- | --- | --- | --- | --- | --- | --- | --- |
| **DOB :** DD/MM/YYYY  **Age:** YY □ Unknown  **SEX:** □ M □ F □ Unknown | | **DATE OF TRAUMA:**  DD/MM/YYYY | | | | | | | | | | |
|  |  | **TIME OF TRAUMA:** | | | | | HH/MM | | | |  | |
|  |  | **DATE OF ARRIVAL:**  DD/MM/YYYY | | | | | | | | | | |
|  |  | **TIME OF ARRIVAL:** | | | HH/MM | | |  | | | | |
|  |  | **MODE OF ARRIVAL:** | | □ **1** FOOT | | | □ **2** BICYCLE | | | | | □ **3** MOTOR CYCLE |
|  |  |  |  | □ **4** THREE -  WHEELER | | | □ **5** TAXI | | | | | □ **6** PUBLIC  TRANSPORT |
|  |  |  |  | □ **7** PRIVATE CAR | | | □ **8** GROUND  AMBULANCE | | | | | □ **9** HELICOPTER  AMBULANCE |
|  |  |  |  | □ **10** FIXED-WING  AMBULANCE | | | □ **11** POLICE | | | | | □ **77** OTHER |
|  |  |  |  | □ **99** UNKOWN | | |  | | | | |  |
|  |  |  |  |  | | | | | | | | |
|  |  |  |  |  | | |  | | | | |  |
| **PRE-HOSPITAL INTERVENTIONS:** | □ NONE □ C-COLLAR □ SPINE BOARD □ SPLINTING □ INTUBATED □ OXYGEN □ ANALGESIA  □ IV ACCESS □ WOUND DRESSING □ OTHER (SPECIFY): | | | | | | | | | | | |
| **MEDICAL HISTORY:** *Check all that apply* | □ NONE | | □ DIABETES | | | □ HYPERTENSION | | | | □ MALIGNANCY | | |
|  | □ HEART DISEASE | | □ STROKE | | | □ ASTHMA/COPD | | | | □ OTHER: | | |
|  | □ PREGNANT | | □ 1^ST^ TRIMESTRE | | | □ 2^ND^ TRIMESTRE | | | | □ 3^RD^ TRIMESTRE | | |
| **PLACE OF INJURY:** | □ **1** PRIVATE HOME  □ **5** RAILWAY  STATION  □ **9** SEA,LAKE,  RIVER ,DAM,  BOREHOLE ,WELL  □ **13** OPEN LAND,  BEACH, FOREST,  DESERT | | □ **2** RESIDENTIAL  INSTITUTION  □ **6** TRADE AREA  □ **10** ATHLETICS AREA  □ **77** OTHER | | | □ **3** MEDICAL  INSTITUTION  □ **7** INDUSTRIAL  AREA  □ **11** SCHOOL  □ **99** UNKNOWN | | | | □ **4** STREET/ HIGHWAY/  ROAD  □ **8** FARM  □ **12** PUBLIC  ADMINISTRATIVE AREA | | |
| **ACTIVITY DURING TRAUMA:** | □ **1** WORK (INCOME)  □ **5** TRAVELLING | | □ **2** OTHER WORK  (NON INCOME)  □ **6** VITAL ACTIVITIES | | | □ **3** SPORTS  □ **77** OTHER | | | | □ **4** LEISURE  □ **99** UNKNOWN | | |
| **INTENT OF ACTIVITY:** | **□ 1** ACCIDENTAL  □ **5** UNDETERMINED | | □ **2** INTENTIONAL  (SELF HARM)  □ **99** UNKNOWN | | | □ **3** INTENTIONAL  (ASSAULT) | | | | □ **4** LEGAL  INTERVENTION | | |
| **MECHANISM OF INJURY:** *Check all that apply* | □ **1** ROAD TRAFFIC  INCIDENT  □ **5** SHARP FORCE  (STAB/ CUT)  □ **11** SUFFOCATION,  CHOKING,HANGING  □ **15** ENVENOMATION  (ANIMAL/PLANT) | | □ **2** OTHER TRANSPORT  INCIDENT  □ **6** SHARP FORCE  (ANIMAL BITE)  □ **12** ELECTROCUTION  □ **77** OTHER | | | □ **3** BLUNT FORCE  □ **7** DROWNING  _____HRS  □ **13** GUN SHOT  □ **99** UNKNOWN | | | | □ **4** FALL  □ **10** POISONING  □ **14** EXPLOSIVE BLAST | | |
|  | □  **8** BURNS  (*smoke/fire/flames*)  □  **9** BURNS  (*contact with*  *heat/scald*) | | □ FLAME  □ RADIATION  □ LIGHTENING | | | □ INHALATION  □ HOT LIQUID | | | □ ELECTRICAL DOMESTIC  □ ELECTRICAL INDSUTRIAL | | | |

| □ **ROAD TRAFFIC ACCIDENT □ N/A** | | | | | | | | | | | | | | | | | | | | | | | | | | | | | | | | | | | | | | | | | | | |
| --- | --- | --- | --- | --- | --- | --- | --- | --- | --- | --- | --- | --- | --- | --- | --- | --- | --- | --- | --- | --- | --- | --- | --- | --- | --- | --- | --- | --- | --- | --- | --- | --- | --- | --- | --- | --- | --- | --- | --- | --- | --- | --- | --- |
| **TYPE OF VEHICLE INVOLVED:** | | □ CAR | | | □ BUS | | | | | | | | | | | | | □ BICYCLE | | | | | | | | | | □ OTHER: | | | | | | | | | | | | | | | |
|  |  | □ MATATU | | | □ MOTOR CYCLE | | | | | | | | | | | | | □ ATV | | | | | | | | | |  |  |  |  |  |  |  |  |  |  |  |  |  |  |  |  |
| **PATIENT WAS:** | | □ DRIVER/RIDER | | | | | | | □ PEDESTRIAN | | | | | | | | | | | □ FRONT SEAT PASSENGER | | | | | | | | | | | | | | | | □ BACK SEAT PASSENGER | | | | | | | |
| **IMPACT PROTECTION:** | | | □ NONE | □ SEAT BELT | | | | | | | | | | | □ AIRBAG | | | | | | | | | □ HELMET | | | | | □ CHILD SEAT | | | | | | | | | | | □ OTHER: | | | |
| □ **ASSAULT □ N/A** | | | | | | | | | | | | | | | | | | | | | | | | | | | | | | | | | | | | | | | | | | | |
| **ASSAILANT:** | □ KNOWN | | | □ UNKNOWN | | | | | | | | | | | □ SPOUSE | | | | | | | | | □ TERRORISM | | | | | | | □ ANIMAL (*SPECIFY*): | | | | | | | | | | | | |
| **PATIENT ASSESSMENT** | | | | | | | | | | | | | | | | | | | | | | | | | | | | | | | | | | | | | | | | | | | |
| **AIRWAY:** | □ PATENT/TALKING | | | | | | | | | | | | | | | □ NEEDS INTUBATION | | | | | | | | | | | | | | | | | | | | | □ INTUBATED | | | | | | |
| **BREATHING:** | □ SPONTANEOUS | | | | | | | □ LABOURED | | | | | | | | | | | □ AGONAL | | | | | | □ NO EFFORT | | | | | | | | | **RATE:____/MIN SPO2:_____%** | | | | | | | | | |
| **RESPIRATORY RATE QUALIFIER:** | □ **1** UNASSISTED RESPIRATORY RATE | | | | | | | | | | | | | | | | | | | | □ **2** ASSISTED RESPIRATORY RATE | | | | | | | | | | | | | | | | | | | | | □ **99** UNKNOWN | |
| **CIRCULATION:** | □ CENTRAL PULSE | | | | | □ PERIPHERAL PULSE | | | | | | | | | | | | | | | | | □ NO PULSE | | | | | | | **RATE:____/MIN BP:____/____MMHG** | | | | | | | | | | | | | |
| **DISABILITY:** | □ AWAKE AND ALERT | | | | | | | | | | □ VERBAL STIMULI ELICITS RESPONSE | | | | | | | | | | | | | | | | □ PAINFUL STIMULI ELICITS RESPONSE | | | | | | | | | | | | | | | □ UNRESPONSVE | |
| **GLASGOW COMA SCALE (GCS):** | **EYE OPENING**  □ **99** UNKNOWN  □ **4** SPONTANEOUS  □ **3** TO VERBAL  □ **2** TO PAIN  □ **1** NONE | | | | | | | | | | | | **VERBAL**  □ **99** UNKNOWN  □ **5** ORIENTED  □ **4** CONFUSED  □ **3** INAPROPRIATE RESPONSE  □ **2** INCOMPREHENSIBLE  □ **1** NONE/INTUBATED | | | | | | | | | | | | | | | | | | | **MOTOR**  □ **99** UNKNOWN  □ **6** OBEYS  □ **5** LOCALIZES PAIN  □ **4** WITHDRAWS FROM PAIN  □ **3** FLEXOR POSTURING  □ **2** EXTENSOR POSTURING  □ **1** NONE/CHEMICALLY PARALYZED | | | | | | | | | | | |
| **GCS (TOTAL):** | □ **15** MAXIMUM | | | | | | | | | □ Number__________ | | | | | | | | | | | | | | | | □ **3** MINIMUM | | | | | | | | | | | | | | | □ **99** UNKNOWN | | |
| **GSC QUALIFIER:** | □ **1** PATIENT CHEMICALLY SEDATED OR PARALYZED  □ **4** VALID GCS | | | | | | | | | | | | | □ **2** OBSTRUCTION TO THE PATIENT’S EYES  □ **99** UNKNOWN | | | | | | | | | | | | | | | | | | | □ **3** PATIENT INTUBATED  **RBS:** ___________mmol/L | | | | | | | | | | |
| **SIGNS OF LIFE** | □ **1** ARRIVED WITH NO SIGNS OF LIFE | | | | | | | | | | | □ **2** ARRIVED WITH SIGNS OF LIFE | | | | | | | | | | | | | | | | | | | □ **77** OTHER | | | | | | | | | | | | □ **99** UNKNOWN |
| **PUPILS** | **LEFT** | | | | | | | | | | | | | | | | | | | | | **RIGHT** | | | | | | | | | | | | | | | | | | | | | |
|  | □ REACTIVE  □ NON-REACTIVE | | | | | | □ DILATED  □ PIN-POINT | | | | | | | | | | | | | | | □ REACTIVE  □ NON-REACTIVE | | | | | | | | | | | | | | | | | □ DILATED  □ PIN-POINT | | | | |
| **ALCOHOL USE** | □ NONE | | | | | | □ PATIENT | | | | | | | | | | | | | | | □ ASSAILANT | | | | | | | | | | | | | | | | □ BOTH | | | | | |
| **TEMPERATURE** | ___________ ^O^C | | | | | | | | | | | | | | | | | | | | | | | | | | | | | | | | | | | | | | | | | | |
| **NATURE OF INJURY:** | □ **1** BRUISE/ABRASION □ **4** DEEP LACERATION  □ **7** OPEN FRACTURE □ **10** CONCUSSION  □ **13** PNEUMOTHORAX | | | | | | | | | | | | □ **2** SPRAIN/STRAIN  □ **5** AVULSION/AMPUTATION  □ **8** DISLOCATION  □ **11** ORGAN SYSTEM INJURY  □ **77** OTHER | | | | | | | | | | | | | | | | | | | | □ **3** SUPERFICIAL LACERATION  □ **6** CLOSED FRACTUE  □ **9** BURN  □ **12** HAEMORRHAGE  □ **99** UNKNOWN | | | | | | | | | | |
| **SEVERITY OF INJURY:** | □ **1** MINOR  □ **4** SEVERE  □ **99** UNKNOWN | | | | | | | | | | | | | | | | □ **2** MODERATE  □ **5** CRITICAL | | | | | | | | | | | | | | | | | | □ **3** SERIOUS  □ **6** MAXIMAL (CURRENTLY UNTREATABLE) | | | | | | | | |

| **LOCATION OF INJURY:** | □ **1** HEAD OR NECK  □ **4** ABDOMINAL AND PELVIS CONTENTS | □ **2** FACE  □ **5** EXTREMITIES INCLUDING BONY PELVIS | □ **3** CHEST  □ **6** EXTERNAL (SKIN)  □ **99** UNKNOWN |
| --- | --- | --- | --- |
| **DESCRIPTION OF INJURIES: HEAD □ NONE**  ***Include exact anatomical site of each injury, it’s gross pathology on examination, US, CT, surgery (for burns include %)*** | | | |
| **INJURIES: NECK □ NONE**  ***Include exact anatomical site of each injury, it’s gross pathology on examination, US, CT, surgery (for burns include %)*** | | | |
| **INJURIES: FACE □ NONE** | | | |
| **INJURIES: CHEST □ NONE**  ***Include exact anatomical site of each injury, it’s gross pathology on examination, US, CT, surgery (for burns include %)*** | | | |

| **INJURIES: ABDOMEN □ NONE**  ***Include exact anatomical site of each injury, it’s gross pathology on examination, US, CT, surgery (for burns include %)*** |
| --- |
| **INJURIES: PELVIS □ NONE**  ***Include exact anatomical site of each injury, it’s gross pathology on examination, US, CT, surgery (for burns include %)*** |
| **INJURIES: GENITALIA □ NONE**  ***Include exact anatomical site of each injury, it’s gross pathology on examination, US, CT, surgery (for burns include %)*** |
| **INJURIES: BACK □ NONE**  ***Include exact anatomical site of each injury, it’s gross pathology on examination, US, CT, surgery (for burns include %)*** |
| **INJURIES: EXTREMITIES □ NONE**  ***Include exact anatomical site of each injury, it’s gross pathology on examination, US, CT, surgery (for burns include %)*** |

| **INJURIES: EXTERNAL(SKIN) □ NONE** | | | | | | | |
| --- | --- | --- | --- | --- | --- | --- | --- |
| **□ UNKNOWN** | | | | | | | |
|  | | | | | | | |
| **EMERGENCY**  **DEPARTMENT**  **DISPOSITION:** | □ **1** WARD  □ **4** DIED PRIOR TO ARRIVAL | | □ **2** OPERATING THEATRE  □ **5** DIED IN EMERGENCY  DEPARTMENT | | | □ **3** ICU | |
| **DATE/TIME OF EMERGENCY DEPARTMENT DEPATURE:** DD/MM/YYYY HH/MM □ **99** UNKNOWN | | | | | | | |
| **HOSPITAL DISCHARGE DISPOSITION:** | □ **1** HOME  □ **4** TRANSFERRED TO ANOTHER HOSPITAL | | □ **2** LEFT AGAINST MEDICAL ADVICE  □ **77** OTHER | | □ **3** DIED IN HOSPITAL  □ **99** UNKNOWN | | |
| **DATE OF HOSPITAL DISCHARGE:** DD/MM/YYYY | | | | | | | |
| **TIME OF HOSPITAL DISCHARGE:** | | HH/MM | |  | | |  |
